# Lower serotonin transporter binding in patients with cervical dystonia is associated with psychiatric symptoms

**DOI:** 10.1186/s13550-017-0338-4

**Published:** 2017-10-25

**Authors:** E. Zoons, J. Booij, J. D. Speelman, Y. E. M. Dreissen, M. Smit, M. A. J. Tijssen

**Affiliations:** 1 Department of Neurology, Academic Medical Center, PO Box 22660, 1100 DD Amsterdam, The Netherlands; 2 Department of Nuclear Medicine, Academic Medical Center, Amsterdam, The Netherlands; 3 Department of Neurology, University Medical Center, Groningen, The Netherlands

**Keywords:** Cervical dystonia, SPECT, Serotonin transporter (SERT), Depression

## Abstract

**Background:**

Cervical dystonia (CD) is often accompanied by depressive symptoms, anxiety, and jerks/tremor. The dopamine transporter (DAT) binding is related with both depressive symptoms and jerks/tremor in CD. Serotonergic and dopaminergic systems are closely related. As serotonin is involved in the pathophysiology of psychiatric symptoms and jerks, we expected an altered serotoninergic system in CD. We hypothesized that CD is associated with reduced serotonin transporter (SERT) binding, more specific that SERT binding is lower in CD patients with psychiatric symptoms and/or jerks/tremor compared to those without, and to controls. The balance between SERT and DAT binding can be altered in different CD phenotypes.

**Results:**

In 23 CD patients and 14 healthy controls, SERT binding in the diencephalon/midbrain was assessed using [^123^I]FP-CIT SPECT, with a brain-dedicated system. The specific to non-specific binding ratio (binding potential; BP_ND_) to SERT was the main outcome measure. There was a clear trend towards reduced SERT BP_ND_ in CD patients with psychiatric symptoms compared to those without (*p* = 0.05). There was no correlation between SERT binding and dystonia, jerks, or anxiety. There was a significant positive correlation between extrastriatal SERT and striatal DAT BP_ND_ in CD patients with jerks, but not in patients without jerks.

**Conclusion:**

CD patients with psychiatric symptoms have lower SERT binding in the midbrain/diencephalon, while dystonia and jerks appear unrelated to SERT binding. The balance between extrastriatal SERT and striatal DAT binding is different in CD with and without jerks.

## Background

Dystonia is a syndrome characterized by sustained or intermittent muscle contractions causing abnormal, often repetitive, movements, postures, or both. Idiopathic cervical dystonia (CD; dystonia of the neck) is the most common form of dystonia [1]. Dystonia has commonly been assumed to be a disorder of the dopaminergic system [2]. Previous imaging studies in different types of dystonia have mostly shown normal radiotracer binding to striatal dopamine transporters (DAT) and normal or decreased binding to striatal dopamine D_2/3_ receptors (D2/3 receptors) [3, 4]. We recently showed normal striatal DAT binding but decreased D2/3 receptor binding in CD. We also showed a significant relationship between striatal DAT binding and jerks/tremor of the head, which is a common symptom in CD, and a significant and negative relationship between both striatal DAT and D2/3 receptor binding and depressive symptoms [5].

Psychiatric symptoms, mainly depressive symptoms and anxiety, are very common in patients with dystonia with an estimated lifetime prevalence between 40 and 70% [6] and are thought to be part of the dystonia phenotype [6]. Single photon emission computed tomography (SPECT) and positron emission tomography (PET) imaging studies in patients with major depression mostly showed reduced serotonin transporter (SERT) binding [7, 8]. Reduced SERT binding in depression is hypothesized to reflect decreased expression of SERTs. In line with this, postmortem studies in patients with major depressive disorder found reduced expression of SERTs in several brain regions including the midbrain [9].

The role of serotonin in dystonia has not been studied with imaging or postmortem, but there are strong indications that alterations of the serotonergic system may indeed play a role. There have been many case reports of drug-induced dystonia after the use of selective serotonin reuptake inhibitors (SSRIs) [10]. Furthermore, studies in patients with dopa-responsive dystonia and idiopathic adult-onset dystonia found decreased levels of serotonin metabolites, mainly 5-hydroxyindoleacetic acid (5-HIAA), in cerebrospinal fluid [11, 12]. In the rare genetic syndrome of 5-HIAA deficiency, serotonin levels are low in the brain and patients suffer from dystonia [13]. In addition, the role of serotonin in myoclonus pathophysiology gained more attention lately [14], and approximately 50% of CD patients have myoclonus (jerks) or tremor of the head [15].

There are strong clues that the brain serotonin and dopamine systems are closely interrelated. In a rat study, long-term treatment with SSRIs led to a significant reduction of tyrosine hydroxylase, an important enzyme in the biosynthesis of dopamine, in the substantia nigra and striatum [16]. Furthermore, dopaminergic neurons contain serotonin receptors, and serotonergic neurons contain dopamine receptors [16]. In an animal model for myoclonus dystonia, an inherited form of dystonia caused by a mutation in the epsilon-sarcoglycan gene (SCGE); the hypothesis of a dysbalanced dopamine-serotonin system has been confirmed. SCGE knockout mice show all the signs of dystonia, from jerks to depression-like behavior. After sacrificing, neurochemical studies in the striata of these mice showed increased levels of dopamine metabolites that correlated with motor performance (dystonia and jerks), while the level of serotonin metabolites, that was not significantly different from wild-type mice, inversely correlated with motor performance [17]. Jerks in CD, that resemble myoclonus in myoclonus dystonia, may also be related to alterations of the serotonin system.

Our hypothesis was that CD is associated with reduced SERT binding, especially in CD patients with psychiatric symptoms. Because of the relation of both dopamine and serotonin metabolites and motor performance, including jerks, in SCGE knockout mice, we expected the balance between DAT and SERT binding to be different in CD patients with jerks compared to those without. We tested these hypotheses by imaging SERT in the diencephalon/midbrain and DAT in the striatum with [^123^I]fluoropropyl-carbomethoxy-3β(4-iodophenyltropane) (FP-CIT) SPECT.

## Methods

### Subjects

Included subjects also participated in another study recently published [5]. In short, we included patients with idiopathic CD with stable disease severity for at least 1 year on the Tsui scale, age between 35 and 80 years, and ongoing treatment with botulinum neurotoxin (BoNT) injections, which is the current standard treatment for focal dystonia. BoNT injections were administered on the day of SPECT scanning or a maximum of 7 days prior to or after scanning. Exclusion criteria were other relevant neurological conditions at inclusion or in the past, treatment with deep brain stimulation (DBS), use of medication with a known dopaminergic or serotonergic effect in the past 6 months, including SSRIs [18], and pregnancy or lactation. Patients were allowed to use other medication including low-dose benzodiazepines (for example clonazepam 1 mg twice daily). Healthy age- and sex-matched subjects with a normal neurological examination and no self-reported (family) history of dystonia, myoclonus, or psychiatric illness recruited through flyers and databases served as the control group. Written informed consent was obtained in all subjects, and the study was approved by the local medical ethics committee.

### Scoring neurological and psychiatric symptoms

The neurological examination of patients was videotaped and blindly scored by two independent clinicians. The Toronto Western Spasmodic Torticollis Rating Scale (TWSTRS) [19] and the Tsui scale were used to score dystonic symptoms [20]. The Unified Myoclonic Rating Scale (UMRS) was used to score jerks [21]. In our previous study [5], we showed good interobserver agreement for the TWSRTS and Tsui (> 0.80 intraclass correlation coefficients) and reasonable agreement for the UMRS (0.73 intraclass correlation coefficients). The average score of the two experts on the Tsui, TWSTRS, and UMRS was used in the statistical analysis. The psychiatric examination consisted of an interview, performed by a trained investigator (EZ; YD). The Mini International Neuropsychiatric Interview (MINI)-Plus and Montgomery-Åsberg Depression Rating Scale (MADRS) were included, as well as several questionnaires concerning symptoms of depression (Beck Depression Inventory (BDI)) and anxiety (Liebowitz Social Anxiety Scale (LSAS) and the Beck Anxiety Inventory (BAI)for anxiety ). The psychiatric interview was performed on the day of the SPECT scan, and questionnaires were answered in the week preceding the SPECT scan. Subjects were judged to have a depressive disorder when they fulfilled the according criteria on the MINI (current depression) and/or had a BDI ≥ 14 points or MADRS ≥ 20 points, consistent with moderate-severe depression. Subjects were judged to have an anxiety disorder when they fulfilled the according criteria on the MINI (current anxiety disorder) and/or had a BAI ≥ 16 points or LSAS ≥ 30 points, consistent with moderate-severe anxiety.

### SPECT imaging

All subjects received 300 mg potassium iodide (three capsules of 100 mg each) to block thyroid uptake of free radioactive iodide before administration of the tracer. Then, all subjects received a mean dose of 100 MBq (2.7 mCi) [^123^I]FP-CIT intravenously (produced according to GMP criteria by GE Healthcare) as a single bolus [22]. Scans were acquired 2 h after bolus injection to optimally visualize extrastriatal SERT binding, a technique that has been validated before [23]. SPECT studies were performed using a 12-detector single-slice brain-dedicated scanner (Neurofocus 810, which is an upgrade of the Strichman Medical Equipment); this system was described extensively by Stoddart and Stoddart [24], with a full-width at half-maximum in-slice resolution of approximately 5–6 mm, throughout the 20-cm field-of-view, and a system volume sensitivity of 0.22 (counts/s)/(Bq/ml) [25]. After positioning of the subjects with the head parallel to the orbitomeatal line, axial slices parallel, and upward from the orbitomeatal line to the vertex were acquired in 5-mm steps. An average of 15 slices with 3.5-min scanning time per slice was acquired in a 64 × 64 matrix. The energy window was set at 140–178 keV. Images were corrected for attenuation, as earlier described, and reconstructed in a 3-D mode using Neurofocus proprietary software (Neurofocus Inc., USA) and the manufacturer’s recommended iterative reconstruction algorithm, which was based on maximum a-posteriori (MAP) reconstruction methods [26]. The 3-D reconstructed images were randomly numbered and analyzed blinded for the subject group (CD patient with/without jerks or control) by one observer (EZ). Fixed regions of interest (ROIs) for the diencephalon and midbrain combined were positioned as earlier described [23, 27]. The four consecutive slices with highest activity in the ROI were pooled together, and average activity was calculated. This was also done for the cerebellum as reference region using the two to three consecutive slices with highest activity. Specific to non-specific binding ratio was calculated as [(activity in ROI − activity in reference region)/activity in reference region], representing the binding potential (BP_ND_) [28]. Test-retest variability of this analysis was calculated in a random subset of subjects (*n* = 10) by using the formula (test-retest)/((test + retest)/2) × 100%. Average variability was 10%, which is comparable to previously published variability in extrastriatal [^123^I]FP-CIT binding in the midbrain [29] and only slightly higher than variability in striatal [^123^I]FP-CIT binding, which is ~ 7.5% [22].

As published before [5], we also measured DAT BP_ND_ in the striatum 3 h after the same [^123^I]FP-CIT injection in the same subjects. We used these values to test the correlation between SERT and DAT BP_ND_.

### Statistical analysis

Mann-Whitney *U* test and Kruskal-Wallis test were used to assess differences in baseline characteristics, SERT BP_ND,_ and SERT to DAT ratio between different groups of subjects. Chi-square and Fisher’s exact test were used to assess dichotomous variables. Linear regression was used to assess if differences in baseline characteristics explained differences in SERT BP_ND_, both between patients with and without jerks/tremor, and between CD patients and healthy controls. Linear regression was also used for assessing relationships between SERT BP_ND_ and motor and psychiatric scores. Since age and sex are known to have an effect on SERT BP_ND_ as measured with [^123^I]FP-CIT SPECT [30], we corrected for these factors. Analyses were carried out using SPSS version 23, and differences were considered significant at *p* < 0.05.

## Results

### Clinical characteristics

We planned to include 29 CD patients and compare them to 15 matched healthy controls. Due to technical difficulties, one [^123^I]FP-CIT scan of a control and six [^123^I]FP-CIT scans of patients had to be removed from the analysis. More specific, not in all scans the whole cerebellum was scanned because of a skewed position of the head (the brain-dedicated system is a tomographic system), and consequently, this region could not be used as a reliable reference region [5]. We could use data from 23 CD patients (12 with jerks/tremor and 11 without) and 14 controls. Baseline characteristics are depicted in Table 1, no significant differences between groups were detected. Low-dose benzodiazepines were used by 6/23 patients: oxazepam or diazepam on as needed base (three patients), oxazepam 5 mg once daily (one patient), and clonazepam 0.5 mg once or twice daily (two patients). In total, 8/11 (73%) of the patients without comorbid jerks/tremor fulfilled the criteria for a psychiatric diagnosis (any diagnosis on the MINI and/or a score above the before mentioned cutoff values on the BDI, MADRS, LSAS, or BAI) compared to 6/12 (50%) of the patients with comorbid jerks/tremor. The most common condition was social anxiety disorder (7/23 patients; 30%). In one patient, both a depressive disorder as well as social anxiety disorder were present. As mentioned in our previous study, one control fulfilled the criteria for alcohol abuse in the past and one control scored 34 on the LSAS meeting the criteria of social anxiety disorder [5].

**Table 1:** Baseline characteristics

Characteristics	CD with jerks (*n* = 12)	CD without jerks (*n* = 11)	Controls (*n* = 14)	*p* value
Age, *y*, median (IQR)	62 (51.5–63.75)	54 (46–62)	61 (55–62)	0.27
Men, *n* (%)	6 (50%)	5 (45%)	7 (50%)	0.97
Tsui, median (IQR)	9 (7.5–13)	7.5 (5–14)	N/A	0.49
TWSTRS total, median (IQR)	16.5 (14.5–21)	14 (13.5–19)	N/A	0.28
UMRS, median (range)	12.5 (7–19)	1 (0.5–2)	N/A	< 0.001
Psychiatric disorders, *n* (%)	6 (50%)	8 (73%)	2 (14%)	0.01
Anxiety disorder, *n* (%)	4 (33%)	6 (55%)	1 (7%)	0.03
Depressive disorder, *n* (%)	2 (17%)	3 (27%)	0 (0%)	0.13
BDI, median (IQR)	4.5 (2.25–9.5)	5 (3–9)	1.5 (0–3)	0.007
MADRS, median (IQR)	2 (0–3.75)	4 (0–9)	0.5 (0–2)	0.03
LSAS, median (IQR)	10.5 (2–33.5)	17 (5–44)	4 (0.75–8.5)	0.03
BAI, median (IQR)	5.5 (3.25–12.25)	4 (1–12)	0.5 (0–1)	0.002

*p* values are depicted for comparisons between the three groups in all cases except for Tsui, TWSTRS, and UMRS, where only the two groups of patients are compared

*BAI* Beck Anxiety Inventory, *BDI* Beck Depression Inventory, *CD* cervical dystonia, *IQR* interquartile range, *LSAS* Leibowitz Social Anxiety Scale, *MADRS* Montgomery-Åsberg Depression Rating Scale, T*WSTRS* Toronto Western Spasmodic Torticollis Rating Scale, *UMRS* Unified Myoclonic Rating Scale

### [^123^I]FP-CIT SPECT—serotonin transporter

Median SERT BP_ND_ was 0.26 in controls (IQR 0.10–0.40) and 0.25 in patients (IQR 0.13–0.40; *p* = 0.89, Mann-Whitney *U* test). In addition, median SERT BP_ND_ was not significantly different in patients with jerks/tremor (0.28; IQR 0.15–0.42) compared to patients without jerks/tremor (0.17; IQR 0.11–0.34; *p* = 0.49, Mann-Whitney *U* test). There was no significant correlation between the diencephalon/midbrain SERT BP_ND_ and scores on Tsui (*p* = 0.79), TWSTRS (*p* = 0.20), or UMRS (*p* = 0.32; linear regression) in CD patients.

Median SERT BP_ND_ was 0.20 in patients with psychiatric co-morbidity (*n* = 14; IQR 0.11–0.28) and 0.42 in patients without a psychiatric diagnosis (*n* = 9; IQR 0.16–0.47; *p* = 0.05, Mann-Whitney *U* test). This trend was not present between patients with any anxiety disorder (*n* = 10; median SERT BP_ND_ 0.24 (IQR 0.15–0.28)) and patients without any anxiety disorder (*n* = 13; median SERT BP_ND_ 0.31 (IQR 0.11–0.44); *p* = 0.45, Mann-Whitney *U* test). There was a trend towards a difference between patients with a depression (*n* = 5; median SERT BP_ND_ 0.14 (IQR 0.10–0.22)) and patients without (*n* = 18; median SERT BP_ND_ 0.28 (IQR 0.15–0.42); *p* = 0.09, Mann-Whitney *U* test). There was no significant correlation between the diencephalon/midbrain SERT BP_ND_ and scores on BDI (*p* = 0.43), MADRS (*p* = 0.43), LSAS (*p* = 0.29), or BAI (*p* = 0.34, linear regression) in CD patients.

Correcting for age and sex by means of linear regression did not change the results in any of our analyses. Regression coefficients and *p* values before and after correction are depicted in Table 2.

**Table 2:** Regression analyses for extrastriatal SERT binding corrected for age and sex

	Regression coefficient before correction	*p* value before correction	Regression coefficient corrected for age and sex	*p* value corrected for age and sex
Patients vs controls (95% CI)	−0.04 (−0.17-0.10)	0.59	−0.03 (−0.18-0.11)	0.62
Jerks vs no jerks (95% CI)	0.04 (−0.10-0.18)	0.55	0.04 (−0.12-0.19)	0.62
Psychiatry vs no psychiatry (95% CI)	−0.13 (−0.26-(−0.01))	0.04	−0.14 (−0.28–(−0.00))	0.05
Anxiety vs no anxiety (95% CI)	−0.08 (−0.21-0.06)	0.24	−0.08 (−0.23-0.07)	0.27
Depression vs no depression (95% CI)	−0.12 (−0.28-0.04)	0.14	−0.14 (−0.31-0.03)	0.10

### DAT and SERT BP_ND_

**Fig. 1:**
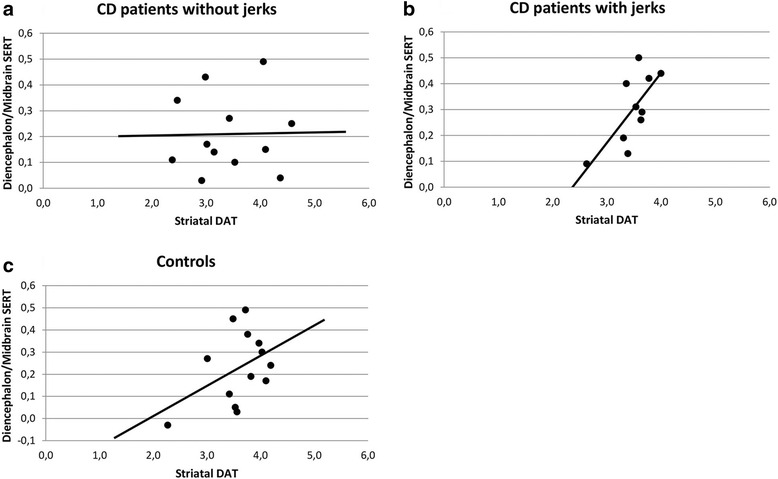
The correlation between striatal DAT BP_ND_ and the diencephalon/midbrain SERT BP_ND_ is shown for the three groups separately. **a** CD patients without jerks, **b** CD patients with jerks, **c** Controls

There was a statistically significant correlation between the diencephalon/midbrain SERT BP_ND_ and striatal DAT BP_ND_ in patients with jerks (R^2^ 0.51, regression coefficient 0.27 (0.06–0.49; *p* = 0.02; see Fig. 1b). Correcting for any psychiatric diagnosis or depression did not change these results significantly. This correlation was not present in patients without jerks (R^2^ 0.04, regression coefficient − 0.11–0.20; *p* = 0.56, see Fig. 1a) or in controls (*p* = 0.14, see Fig. 1c).

## Discussion

This study did not show significant differences in the diencephalon/midbrain SERT binding in patients with idiopathic CD compared to controls. However, we did find a trend (*p* = 0.05) towards decreased diencephalon/midbrain SERT BP_ND_ in CD patients with psychiatric comorbidity and in CD patients with a depression compared to those without. In this group, results failed to reach significance, likely due to the small number of CD patients with depression (*n* = 5). Furthermore, a significant correlation between the diencephalon/midbrain SERT and striatal DAT binding in CD patients with jerks was detected. This correlation was not present in controls and CD patients without jerks.

A trend towards reduced diencephalon/midbrain SERT BP_ND_ in CD patients with depression is consistent with results previously reported in patients with major depressive disorder, but without dystonia [9]. Reduced SERT binding can be caused by reduced expression of SERTs on serotonergic neurons and/or increased occupancy of the SERT by high levels of endogenous synaptic serotonin. Considering that postmortem studies in major depression found reduced numbers of SERTs in the midbrain, the first theory seems more likely. Furthermore, PET studies in depression have also showed increased binding to serotonin 1_A_ receptors, both the postsynaptic receptors on target neurons throughout the brain as well as the presynaptic autoreceptors in the raphe nuclei. This is in both cases hypothesized to reflect increased number of receptors. The higher number of autoreceptors in the raphe nuclei results in a lower firing frequency and thus reduced synaptic release of serotonin [31]. If this is also the case in patients with CD, this would explain the reduced level of serotonin metabolites that was found in cerebrospinal fluid of dystonia patients, and supports also our postulate that the currently observed lower SERT binding is not caused by an increased release of serotonin [11]. It would be interesting to confirm this theory in a future study in CD with a selective serotonin 1_A_ receptor PET tracer such as [^11^C]WAY-100635.

We found a statistically significant and positive correlation between striatal DAT and extrastriatal SERT BP_ND_ in CD patients with jerks, that was not present in patients without jerks. This might indicate that the relationship between SERT and DAT binding influences the motor phenotype of CD, more specifically whether a patient develops jerks or not. Recent studies in Parkinson’s disease (PD) have shown that the relative amount of DATs and SERTs can be imbalanced and lead to complications. After 5–10 years, patients with PD often develop levodopa-induced dyskinesias (LIDs), and these LIDs are associated with relatively intact serotonergic nerve terminals. PD patients with more available serotonin nerve terminals are more likely to develop LIDs [32]. In agreement with this hypothesis, the striatal SERT/DAT ratio was higher in PD patients suffering from LID than without [33]. In our patients with jerks, higher SERT BP_ND_ was related to higher DAT BP_ND_ which might indicate that CD patients with jerks also have more intact serotonergic nerve terminals. This hypothesis should be tested in future studies, as has been suggested for the development of LIDs in PD [33].

This study has several limitations [5]. We did not obtain data on disease duration. These data are difficult to obtain, as CD often starts with mild complaints and it can take months to years before the right diagnosis is made. Furthermore, psychiatric symptoms, which are now considered part of the phenotype, often precede motor symptoms, making it difficult to establish exactly the moment the disease started [6]. Patients in our study were not allowed to use any dopaminergic or serotonergic medication that may influence [^123^I]FP-CIT binding in vivo [18]; however, they received BoNT injections and were allowed to use benzodiazepines. Furthermore, most subjects used medication for other conditions. Patients were scanned within a week of BoNT administration, making it unlikely that BoNT had an effect on SERT binding ratios. Also, no significant effect of benzodiazepines on [^123^I]FP-CIT binding has been described. Patients in our study used a low dosage of oxazepam or clonazepam. We do not think this has influenced our results. [^123^I]FP-CIT is metabolized by cytochrome P450 type 3A (CYP3A) in the liver, which metabolizes most drugs. Therefore, many drugs might influence [^123^I]FP-CIT metabolism and possibly SERT binding (see discussion in Booij and Kemp [18]). The only potential influence we found was codeine, which was used by one of our patients in a low dose, making it unlikely this influenced our results. Furthermore, this subject had a SERT BP_ND_ of 0.23 which is around the group median.

The radiotracer we used is not a selective radiotracer for SERT. [^123^I]FP-CIT binds both to striatal DAT and extrastriatal SERT, while, for example, [^123^I]ADAM is highly SERT-selective. We chose to use [^123^I]FP-CIT in this study so we could image both striatal DAT and extrastriatal SERT in one scanning session, limiting the number of visits and the exposure to radiation for our participants. Imaging the SERT in extrastriatal brain areas with [^123^I]FP-CIT SPECT is well validated and done before by several groups in several different patient populations [30, 34]. Moreover, in this study, extrastriatal SERT has been examined 2 h after bolus injection, which has been validated previously, and offers the advantage of relatively high count statistics [23]. One factor to take into account is the small, but not negligible, number of serotonergic neurons, and consequently SERT, in the cerebellum, which was used as a reference region, and consequently, we feel it is unlikely that using the cerebellar binding to assess non-specific binding has significantly influenced our results.

SERTs are highly present in the diencephalon/midbrain but are also present in striatum. SERTs in the striatum are highly outnumbered by DATs making it impossible to measure SERT in the striatum with [^123^I]FP-CIT. In future studies, it may be of interest to use selective SERT tracers to evaluate whether our present observations on the diencephalon/midbrain SERT binding are also true for striatal SERT binding. Another limitation of this study is the significant number of scans that had to be excluded from the analysis. Taking this in regard, we still have the largest SPECT imaging study in patients with dystonia thus far, as well as the first study to investigate SERT binding in CD.

## Conclusions

In conclusion, we are the first to show that psychiatric symptoms in CD patients are associated with a trend towards lower extrastriatal SERT binding, while dystonia itself is not related to lower extrastriatal SERT binding. Patients with a relatively intact serotonin system and with a significant correlation between SERT and DAT binding might be more likely to develop jerks, a hypothesis to be tested in future longitudinal studies.

## Abbreviations

[^123^I]FP-CIT: [123I]fluoropropyl-carbomethoxy-3β(4-iodophenyltropane)
5-HIAA: 5-hydroxyindoleacetic acid
BAI: Beck Anxiety Inventory
BDI: Beck Depression Inventory
BoNT: Botulinum neurotoxin
BPND: Non-displaceable binding potential
CD: Cervical dystonia
D2/3 receptor: Dopamine D_2/3_ receptor
DAT: Dopamine transporter
DBS: Deep brain stimulation
IQR: Interquartile range
LIDs: Levodopa-induced dyskinesias
LSAS: Liebowitz Social Anxiety Scale
MADRS: Montgomery-Åsberg Depression Rating Scale
MINI-Plus: Mini International Neuropsychiatric Interview-Plus
PD: Parkinson’s disease
PET: Positron emission tomography
SCGE: Epsilon-sarcoglycan
SERT: Serotonin transporter
SPECT: Single photon emission computed tomography
SSRI: Selective serotonin reuptake inhibitor
TWSTRS: Toronto Western Spasmodic Torticollis Rating Scale
UMRS: Unified Myoclonic Rating Scale
